# Ethanol is indispensable for virucidal hand antisepsis: memorandum from the alcohol-based hand rub (ABHR) Task Force, WHO Collaborating Centre on Patient Safety, and the Commission for Hospital Hygiene and Infection Prevention (KRINKO), Robert Koch Institute, Berlin, Germany

**DOI:** 10.1186/s13756-022-01134-7

**Published:** 2022-07-06

**Authors:** Axel Kramer, Mardjan Arvand, Bärbel Christiansen, Stephanie Dancer, Maren Eggers, Martin Exner, Dieter Müller, Nico T. Mutters, Ingeborg Schwebke, Didier Pittet

**Affiliations:** 1 German Commission for Hospital Hygiene and Infection Prevention at the Robert-Koch Institute, Berlin, Germany; 2 WHO Task Force Alcohol-Based Hand Rub, Zürich, Switzerland; 3 Institute of Hygiene and Environmental Medicine University Medicine Greifswald, Walther-Rathenau-Straße 38, 17475 Greifswald, Germany; 4 Division Hospital Hygiene, Infection Prevention and Control, Robert-Koch Institute, Berlin, Germany; 5 German Commission for Hospital Hygiene and Infection Prevention at the Robert-Koch Institute, Berlin, Germany; 6 Department of Hospital Hygiene, University Hospital Schleswig-Holstein, Lübeck, Germany; 7 Department of Microbiology, University Hospital Hairmyres, Glasgow, UK; 8 School of Applied Sciences, Edinburgh Napier University, Edinburgh, UK; 9 Labor Prof. Dr. G. Enders MVZ GbR, Stuttgart, Germany; 10 Institute of Hygiene and Public Health, University Hospital, Bonn, Germany; 11 Department of Occupational Medicine, University Medical Center Göttingen, Göttingen, Germany; 12 German Association for the Control of Virus Diseases (DVV e. V.), Berlin, Germany; 13 Infection Control Program and WHO Collaborating Centre on Patient Safety, University of Geneva, Hospitals and Faculty of Medicine, Geneva, Switzerland

**Keywords:** Biocidal product regulation, WHO, Hand antisepsis, Ethanol based hand rub, Inactivation, Non-enveloped viruses, Risk–benefit-assessment, Absorption, Worker safety, Patient safety, Memorandum

## Abstract

**Background:**

The approval of ethanol by the Biocidal Products Regulation has been under evaluation since 2007. This follows concern over alcohol uptake from ethanol-based hand rubs (EBHR). If ethanol is classified as carcinogenic, mutagenic, or reprotoxic by the European Chemicals Agency (ECHA), then this would affect infection prevention and control practices.

**Aim:**

A review was performed to prove that ethanol is toxicological uncritical and indispensable for hand antisepsis because of its unique activity against non-enveloped viruses and thus the resulting lack of alternatives. Therefore, the following main points are analyzed: The effectiveness of ethanol in hand hygiene, the evidence of ethanol at blood/tissue levels through hand hygiene in healthcare, and the evidence of toxicity of different blood/tissue ethanol levels and the non-comparability with alcoholic consumption and industrial exposure.

**Results:**

EBHR are essential for preventing infections caused by non-enveloped viruses, especially in healthcare, nursing homes, food industry and other areas. Propanols are effective against enveloped viruses as opposed to non-enveloped viruses but there are no other alternatives for virucidal hand antisepsis. Long-term ingestion of ethanol in the form of alcoholic beverages can cause tumours. However, lifetime exposure to ethanol from occupational exposure < 500 ppm does not significantly contribute to the cancer risk. Mutagenic effects were observed only at doses within the toxic range in animal studies. While reprotoxicity is linked with abuse of alcoholic beverages, there is no epidemiological evidence for this from EBHR use in healthcare facilities or from products containing ethanol in non-healthcare settings.

**Conclusion:**

The body of evidence shows EBHRs have strong efficacy in killing non-enveloped viruses, whereas 1-propanol and 2-propanol do not kill non-enveloped viruses, that pose significant risk of infection. Ethanol absorbed through the skin during hand hygiene is similar to consumption of beverages with hidden ethanol content (< 0.5% v/v), such as apple juice or kefir. There is no risk of carcinogenicity, mutagenicity or reprotoxicity from repeated use of EBHR. Hence, the WHO Task Force strongly recommend retaining ethanol as an essential constituent in hand rubs for healthcare.

## Introduction

The Biocidal Products Regulation regulates the market entry and use of biocidal products. This consists of two stages: 1. approval of a biocidally active substance for certain types of products within the framework of a European procedure, and 2. authorization of a biocidal product at the national or EU level. According to the CLP Regulation (EU) No 1272/2008, biocidal active substances shall normally be subject to the provisions on harmonized classification and labelling [1]. Unless authorised in accordance with Regulation No 528/2012 [2], biocidal products should neither be made available on the market nor used. Approval of ethanol as an active substance has been under evaluation by the responsible Greece authority since 2007 (Table 1). The first decision on classification from the Board was the proposal that ethanol might be classified as ‘’CMR’’ (carcinogen; mutagen; and reprotoxic). In the “Registry of intention” on classification and labelling (CLH), the Greek authority updated a harmonized classification and labelling of ethanol on 27 July 2020 [3]. The current intention of extending harmonized classification provides, inter alia, a classification as reproductive toxicity category 2 (“suspected to have CMR potential for humans”) (Table 1). This is clearly a downgrade from the classification as carcinogenic category 1A and reproductive toxicity 1A (“known to have CMR potential for humans, based largely on human evidence”). However, it is important to note that the Risk Assessment Committee (RAC) of ECHA is not bound by the proposed classification, so that classifying ethanol as carcinogenic and/or reproductive toxicity category 1 by the ECHA still cannot be excluded. Category 1 classification means that ethanol would be marketable as a so-called substitution candidate for a maximum of 5 years. CMR substances of categories 1A and 1B may only be used or placed on the market after the corresponding transitional regulations (sunset date) have been approved by the European Commission according to specific use. Manufacturers, importers and downstream users must apply for these approvals exclusively from the ECHA. In addition, possible alternatives must be analyzed for technical and economic substitutability. The aim is to gradually replace “Substances of Very High Concern” with suitable alternatives.

**Table 1:** State of approval of ethanol as a biocide by Biocidal Products Regulation

Stages of development	Aim or conclusion
2007: Beginning of the evaluation by the Greek authority	Development of a dossier for the European Chemicals Agency (ECHA) as basis for harmonized classification of ethanol
2015: First decision of Greek authority	Classification as CMR: carcinogen; mutagen; reprotoxic
July 2020: Updated decision of Greek authority [3]; not yet finalized for the ECHA	Classification as reproductive toxicity category 2 (presumed human reproductive toxicant)

The CLH dossier from 27 July 2020 should have been submitted to the European Chemicals Agency (ECHA) by 31 December 2020, but this has not yet happened. A final decision on classification from the Board is expected soon.

In October 2020, the German Association for Applied Hygiene published a comment supported by the Federal Association of Public Health Physicians, German Society of Hygiene & Microbiology, German Society for Hospital Hygiene, German Association for Control of Virus Diseases, German Veterinary Society, European Committee on Infection Control, German Virology Society, German Society for Hygiene, Environmental and Preventive Medicine, Austrian Society for Hygiene, Microbiology and Preventive Medicine and Robert Koch Institute, which culminated in the following conclusion:

Ethanol is indispensable as a biocidal product for hygienic hand antisepsis due to its efficacy against non-enveloped viruses, because no alternative exists [4].

The following memorandum of the alcohol-based hand rub (ABHR) Task Force, the WHO Collaborating Centre on Patient Safety and the Commission for Hospital Hygiene & Infection Prevention (KRINKO), supported by the Working Group of Hospital & Practice Hygiene and Working Group of (German) Scientific Medical Societies (AWMF), pursues 2 goals:

- Development of a comprehensive risk–benefit assessment with refutation of classification of ethanol as reproductive toxicity category 2 and justification of the retention of ethanol for hand antisepsis due to the lack of alternatives for inactivation of non-enveloped viruses,
- to draw attention to the possible consequences for infection prevention in healthcare settings if the currently proposed classification as “reproductive toxicity category 2” is chosen.

### Health-related and economic consequences of the proposed classification of ethanol as reproductive toxicity category 2

This EU classification would lead to an international distortion of competition. Companies in non-EU Member countries would be able to produce, use and market EBHR without CMR classification and relevant restrictions. Such classification would also contradict the recital (4)5 of the CLP Regulation, which anticipates a benefit for companies from global harmonization of classification and labelling.

A simple, patent-free hand rub formulation with 80% (v/v) ethanol was recommended in 2009 by the WHO for low-income countries, since it is a less expensive alternative to commercially available preparations and can be produced locally [5]. Of course, WHO formulations could continue to be produced and used outside the EU. But if the marketability in the EU were no longer an option, it is implied that people living in countries outside the EU would be exposed to a risk which the EU excludes for its citizens. This is tantamount to discrimination.

### Specific value of ethanol for virucidal hand antisepsis

Non-enveloped viruses have a significantly higher stability towards chemical agents than enveloped viruses. Of the three alcohols used in hand rub formulations (ethanol, 2-propanol and 1-propanol), only ethanol-based formulations proved to be effective within 30–60 s against non-enveloped viruses such as adeno-, polio-, human entero-, human papilloma-, polyoma-, echo- and coxsackie viruses in quantitative suspension assays [12–16]. Formulations containing 2-propanol and 1-propanol are not sufficiently active (Table 2). 1-propanol was not effective against coxsackie, poliovirus and human enterovirus 71 at a 90% concentration within 5 min [16]. Within 2 min, 80%, 90% and 97% 1-propanol and 2-propanol, and within 3 min, 70% and 90% 1-propanol and 2-propanol, were ineffective against poliovirus [personal communication, Schwebke I]. In contrast, ethanol was effective in concentrations of ≥ 70% in suspension and in vivo on hands [17]. One way to improve the activity of ethanol is to add acids, so that formulations based on 45%, 55%, 60%, 69.4% and 73.5% ethanol (w/w) achieve sufficient activity against poliovirus type 1 within 30 s or 1 min [18–22]. Ethanol activity against polyomavirus SV 40, as surrogate of papillomaviruses, can also be enhanced by adding acids [19, 22]. No comparable findings are available for 2-propanol or 1-propanol [23]. Since ethanol is more effective against non-enveloped viruses, it was chosen as the positive control for assessing the efficacy of hand rubs against viruses on artificially contaminated hands in prEN 17,430:2019 [24].

**Table 2:** Efficacy of solutions based on ethanol and 2-propanol from quantitative suspension tests against non-enveloped viruses (sufficient efficacy is defined for ≥ 4 log_10_ reduction or up to test detection limit)

Species	Strain, type	Alcohol content/necessary exposure time		References
		Ethanol	2-propanol	
Adenovirus	Type 3	77–83%^c^/1 min	90%^b^/5 min^d,e^	[16, 25]
	Type 5	80^a^/30 s		[17]
	Type 7	77–83%^c^/1 min		[25]
	Type 8		70%^c^/2 min^e^	[26]
Coxsackievirus	A16	90%^c^/5 min^d^	90%/60 min^d,e^	[16]
	B1	60%^b^/10 min^d^	95%^c^/10 min^d,e^	[27]
	B2	85%^c^/30 s ^Ø^	90%^c^/1 h^e^	[28]
	B3	90%^c^/15 sec^f^	90%^c^/1 h^e^	
	B4	60%^c^/100 s	90%^c^/1 h^e^	
	B5	77–83%^b^/1 min, 90%^b^/10 s, 80%^b^/30 s	90%/60 min^d,e^	[16, 25]
Echovirus	Type 6	50%^c^/10 min^d^	90%^c^/10 min^d^	[27]
	Type 7	80%^c^/1 min, 90%^c^/30 s	90%/60 min^d,e^	[16]
	Type 11	95%^b^/20 s–1 min, 80%^b^/90%^b^/15 min^d^	90^b^%/15 min^d,e^	[29–31]
	Type 12	70%^b^/80%^b^, 90%^b^/15 min^d^	90%^b^/ 15 min^d,e^	[30]
Feline Calicivirus	Strain F9 (ATCC VR-782)		50%^b^/ 70%^b^, 90^b^/5 min^d,e^	[32]
Human enterovirus	Type 70	70%, 80%, 90%^c^/10 s	70%, 80%, 90%^b^/60 min^d^	[16]
	Type 71	80%^b^/1 min, 90%^b^/30 s	90%^b^/60 min^d,e^	[16, 21]
Hepatitis A virus (HAV)	HM175/24a	80–95%^a^/2 min		[33]
Murine Norovirus	Strain S99 Berlin	80%/30 s 72.4%^b^ (6 mL)/30 s, 86%^b^ (3 mL)/30 s, 89.5^b^ (3 mL)/15 resp. 30 s		[17, 20]
	Type 1	70–90%^b^/ 30 s	70%^b^/5min^d^	[1]
Poliovirus	Type 1	73.5%^a^/10 min^d^, 80%^c^/30 s, 80%^b^/1 min, 90%^c^/10 s, 90%^b^/1 min, 95%^a^/30 s	70%^b^ / 10 min^d,e^ 80%^b^ and 95%^b^ / 10 min^d,e^ 60–90%^b^/15 min^d,e^, 90%/60 min^d,e^	[16, 20, 21, 27, 30, 34–36]
Polyomavirus	SV 40	78.2%^a^/10 min^d,e^		[37]
	HPV16 pseudo-virus	60%, 70%^c^/5 min^d^	60%, 70%^c^/5 min^d,e^	[38]
Rhinovirus	Type 2	80%^c^/3–60 min^d,e^		[39]
Rotavirus	Wa	85%^a^/30 s		[40]

^a^w/w

^b^v/v

^c^Unclear whether w/w or v/v

^d^The exposure time is not feasible in practice

^e^Non-active concentration contact time ratio

^f^3log_10_ reduction

Virucidal hand antisepsis is necessary to interrupt cross-infection with non-enveloped hydrophilic viruses [41, 42] as well as inhibit fomite transmission, which occurs in both gastrointestinal and respiratory infections [43, 44]. This is also the case for norovirus outbreaks on cruise ships [45]. Nosocomial and foodborne outbreaks may be controlled by virucidal hand antisepsis in combination with virucidal surface disinfection (i.e. [46–49]).

While ABHR are standard hygiene procedures in healthcare, recommendations on hand hygiene for the food sector prioritize hand washing, since wet and/or soiled hands may reduce the efficacy of alcohol in inactivating pathogens. Since norovirus is responsible for most (58%) cases of foodborne illness of known etiology [50], causing 125 million (95% UI 70–251 million) cases globally [51], there have been studies to compare hand washing vs ABHR. There is clear evidence to support modifying the FDA Model Food Code to allow the use of EBHR as an alternative to hand washing when heavy soiling is absent [52]. Indeed, medical staff consider hand washing to be inadequate for hand hygiene, especially for bacterial pathogens. Organisms such as *Escherichia coli, Pseudomonas aeruginosa* and *Staphylococcus aureus* are only reduced by 2–2.5 log levels after washing with soap [8]. Only ABHR hand sanitizers are active against murine norovirus (MNV), both in vitro and in vivo [53]. Study antimicrobial soaps could not inactivate MNV [35]. Another in vivo hand-wash study with MNV, conducted according to EN 1499, found soap with CHG 4% to be less effective than handwashing with plain soap [54]. Comparable studies using feline calicivirus, a surrogate virus for human noroviruses, led to a reduction of 1.26 log using liquid soap [55]. Another study showed that EBHR containing 72.4% v/v ethanol was only effective against norovirus after 30 s at twice the volume (6 ml), while a high-ethanol content product (89.5% v/v) was effective after 15 s/3 ml [17]. Handwashing was better than EBHR in one study [56], but the effect was only mechanical. For viral inactivation, it is necessary to use virucides to interrupt further spread. When using virucidal hand rubs as no-rinse formulations, the virus is inactivated directly on hands and the risk of cross-infection is eliminated [53]. Therefore, indication-based use of EBHR is also considered necessary in the food service industry: "It should be possible to differentiate between times when a traditional full handwash (10–15-s scrub followed by rinsing under warm water and drying) must be performed, and when alternative methods, such as a brief hand rinse under warm water, use of a disposable alcohol-based hand wipe, or use of an ethanol-based hand rub, might suffice “ [57].

Considering the contact frequency of staff during patient care, even for isolated patients, and the need for hand antisepsis after each contact with the patients and their surroundings, repeated soap washing is associated with high risk for irritant dermatitis. Therefore, hand washing should be performed only when hands are visibly soiled or contaminated, especially with blood or other body fluids[7]. While dispensers for ABHR can be installed anywhere, access should be restricted in paediatric and psychiatric departments for obvious reasons.

Should a non-enveloped virus, instead of enveloped SARS-CoV-2, cause another pandemic, there would be no sufficiently effective ABHR to prevent transmission if EBHRs with activity against non-enveloped viruses are unavailable.

Table 3 lists examples of non-enveloped viruses that have been detected on hands and for which transmission via hands has led to nosocomial infections and outbreaks. If there is an infection due to one of these viruses, only hand antiseptics based on ethanol 95% or with reduced ethanol content and synergistic additives can be used to interrupt potential transmission.

**Table 3:** Examples of non-enveloped virus transmission via hands leading to nosocomial outbreaks and examples of hand rub efficacy in animal husbandry

Virus	Transmission mode	References
Adenoviruses species A–C, E–G, Species D types 8, 37, 53, 54, 64	Respiratory transmission as well as via hands and surfaces Epidemic keratoconjunctivitis transmission predominantly via hands and surfaces; types 4 and 7 also via swimming pools	[58, 59] [58, 60]
Aphthovirus	This foot-and-mouth virus may be transferred between animals without virucidal hand rub via animal care personnel	[61]
Bocavirus	Mainly respiratory transmission, but also via hands and surfaces	[62]
Coxsackievirus group A types 1–22, 24, group B types 1–6	Transmission mainly via hands; hand, foot and mouth disease worldwide more than 100 outbreaks in nurseries, but also nosocomial infections in adults;	[63–65]
Echovirus serotypes 1–9, 11–21 24–27, 29–33	Transmission predominantly via direct or indirect oral contact, but also via hands	[66]
Enterovirus A 71; D28, 70	Respiratory transmission and through hands; A 71 epidemic paralysis	[62, 64]
HAV	Transmission predominantly fecal–oral via hands by contact with an infected person in cases of inadequate hand antisepsis and inadequate terminal cleaning	[42, 67–78]
HEV	Transmission predominantly fecal–oral via hands, spread is proven through person-to-person contact in genotype-1 infections	[79, 80]
Human papillomavirus	Mainly sexual transmission, but also via hands and vaginal ultrasound probes	[81–84]
Human rhinovirus	Mainly respiratory transmission, but also via hands, especially in an outbreak	[62, 85]
Norovirus	In 18.5% of analyzed outbreaks, virus transmission by HCW via hands	[86]
	After primary food-borne outbreak, further transmission by staff	[87]
	Interruption of an outbreak after changing the virucidally active ingredients	[46]
Parvovirus B19	Mainly respiratory transmission, but also via hands, transmissibility in outbreak 31%	[88, 89]
Rotavirus	Outbreak in adults with transmission between patients via hands	[90]
Poliovirus	Transmission fecal–oral via hands and/or droplets or aerosols	[91]

Since non-enveloped viruses such as HAV and rotaviruses retain infectivity on the skin for up to 6 h [92], it is not surprising that spread has been confirmed experimentally (Table 4).

**Table 4:** Transmission rate from artificially contaminated hands or fingertips to inanimate surfaces

Virus	Level of contamination	Contact time (sec)	Transmission rate	References
HAV	10^4^ PFU	10	After 20 min of drying 24% of input virus transferred (3483 PFU), after 4 h 1.6% (50 PFU)	[15]
Norovirus	150 µL contaminated fecal sample, 1:5 diluted in phosphate-buffered saline (PBS)	10	100% (positive samples after 1st-4th contact) 75% (positive samples after 5th-6th contact) 25% (positive samples after 7th contact)	[93]
	100 µL 10% fecal suspension in PBS (10^6^ PCR detectable units) on fingertip of glove, 1 h drying	Few	from left gloved hand 100% transfer, from right gloved hand 76%	[94]
Rhinovirus	10 µL 10^4^–10^5^ PFU, 20 min drying	5	0.7%-0.9% of inoculum (minimum 190 PFU)	[95]
Rotavirus	10 µL 10% fecal suspension in PBS (10^4^–10^5^ PFU), 20 or 60 min drying	10	16.1% or 2.6% of inoculum	[96]

*PFU* plaque forming unit

### Toxicological evaluation of EBHR use

Evidence for a reprotoxic effect of ethanol originates from experiences with the consumption of alcoholic beverages by pregnant women [97]. There is no epidemiological foundation of toxicity for workers from handling ethanol-containing chemical products in industrial workplaces or from the use of EBHR in healthcare facilities or of ethanol-containing products in the non-healthcare setting. Neither is accidental oral ingestion of EBHR a hazard for healthcare workers.

For 1-propanol and 2-propanol in ABHR, unlike ethanol, there is no suspicion of toxicity from excessive consumption, including carcinogenesis, because neither of these agents are consumed as alcoholic beverages. Because of distinct differences between the three alcohols in terms of metabolically-mediated physiological blood levels, the increase of alcohol blood level above baseline after EBHR was about 157-fold, but after use of 1- and 2-popanol it increased > 1.800- and > 10.000-fold, respectively (Table 5). At the same, in volunteers before the use of ABHR 91 of 107 blood samples (85%) for propan-1-ol, 67 of 107 samples (62.6%) for propan-2-ol and 86 of 107 (80.4%) for ethanol were below the detection limit of 0.13 mg/L, 0.03 mg/L and 0.14 mg/L respectively [102]. The median maximum blood concentration of 11 mg/L after the simulated worst case by 10 surgical hand rubs within 80 min underlines the safety of using EBHR (Table 5).

**Table 5:** Increase of alcohol blood level

ABHR based on	Application	Median maximum blood level (mg/L)	Baseline (mg/L) for each alcohol [98–101, 103]	Increase above baseline	References
Ethanol	10 surgical hand rubs (each 3 min) within 80 min	11	0.07	157-fold	[102]
1-Propanol 70% w/w		18	< 0.01	> 1,800-fold	[103]
2-Propanol 63% w/w		10	< 0.001	> 10,000-fold	

### Absorption after hand rub with ABHR

After excessive exposure (Table 6), only 0.5% to 2.3% of applied ethanol is absorbed, with the highest median blood levels identified between 6.9 and 30.1 mg/L [103]. These findings are in line with the results observed by Miller et al. [104], where five subjects repeatedly (50 times over 4 h) applied 5 mL of an EBHR (62% denatured ethyl alcohol) to both hands and rubbed until dry. The blood ethanol level was < 50 mg/L for all 5 study participants.

**Table 6:** Ethanol concentrations in the blood in relation to exposure according to [103]

Exposure	Ethanol content in the formulation	Volunteers (n)	Median Baseline (mg/L)	Median maximum blood level (mg/L)^a^
20 applications of 4 ml hand rub within 30 min, each application 30 s (hygienic hand antisepsis)	95% w/w (gel)	12	0.11	20.95
	85%	12	0.07	11.45
	55%	12	0.07	6.9
10 applications of 4 ml hand rub, each application 3 min with 5 min break until next application (surgical hand preparation)	95% w/w (gel)	12	0.07	17.5
	85%	12	0.07	30.1
	55%	12	0.07	8.8

^a^After the last application, the median ethanol concentration in peripheral blood increased gradually and peaked after 30 or 20 min; the highest median is given

To clarify potential adverse effects on the pancreas after hand antisepsis by teaching nurses, the following scenario was considered: total rubbing of hands (3 mL of 70% EBHR) consisting of 30 persons 3 times/day in the same room (116 cm^3^) over a period of 48 h interrupted by one night (90 hand rubs/day in the room). Blood ethanol concentrations were predicted using a physiologically-based pharmacokinetic model, which allowed simulation of inhalation and dermal exposure. In the worst-case scenario, the simulation showed that the maximum blood ethanol concentration predicted-5.9 mg/L [104]-is of the same order of magnitude as endogenous ethanol concentration (mean = 1.1 mg/L; median = 0.4 mg/L; range = 0–35 mg/L) in humans without prior alcohol consumption [105].

In comparison, a glass of beer contains about 12 g of ethanol [106], which corresponds to a blood level of 150–250 mg/kg for a 70-kg adult, and produces a peak blood ethanol concentration of 250 mg/L. Fruit juices may contain up to 3 g ethanol per L, and apple juice contains 1 g ethanol per 500 mL [106]. Assuming an absorption rate of 90%, drinking 500 ml of apple juice will result in a blood alcohol concentration of about 85 mg/L ethanol in a 75-kg man or 125 mg/L ethanol in a 60-kg woman [103]. Non-alcoholic beer, flavored water, and orange juice result in blood ethanol levels similar to those seen after hand antisepsis, or even higher [106]. The concentration of ethanol naturally produced by intestinal bacteria is 1.1 mg/L [102, 107] so internal amounts of ethanol resulting from topical application of EBHR are in the range of those associated with consumption of non-alcoholic beverages, which are considered safe for consumers [108].

These results show that there is no significant risk of developmental or reprotoxicity from repeated occupational exposures and/or high frequency use of EBHR [108].

The use of EBHR induces measurable concentrations of ethanol and its metabolite ethyl glucuronide (EtG), a marker of ethanol consumption, in urine, but the measured concentrations are below any harmful or toxic levels [109]. The results demonstrate that dermal and inhaled ethanol absorption from the use of EBHR alone caused mean urinary ethanol concentrations that, on average, were over 60 times lower than those from permitted use of alcohol-containing drinks, food, or cosmetic products. In the United States, harmful intake of ethanol is defined as more than one alcoholic drink equivalent, defined as containing 14 g of pure alcohol (0.33 L of beer or 0.1 L of wine) for women and 18 g for men per day [110, 111]. Germany, Austria, and Switzerland regard the maximum harmless alcohol intake to be 10 g/day for healthy women and 20 g/day for healthy men [112]. Compared with consumption of alcoholic beverages or exposure to consumer products containing ethanol, the amount of ethanol absorption resulting from EBHR application is negligible. In practice, there is no evidence of any harmful effect from using EBHR as a clinical necessity. The following results were confirmed in a double-blind, randomized phase I experimental study: 20 ml of hand rub (74.1% ethanol content) were applied on a 200-cm^2^ gauze swab on skin areas; the gauze swab remained on the skin for 10 min (0.1 mL/cm^2^ treated skin area). Comparing ethanol concentrations at baseline, and after 15 and 60 min, no significant differences could be detected. The detection limit was defined as the ethanol concentration producing an area 3 × larger than the blank area under the curve (0.5 mg/L) [113].

## Animal experiments

After oral administration in mice, there was no fetotoxic or teratogenic effect at 15,000 mg ethanol /kg. It takes 25,000 mg/kg to elicit a fetotoxic and teratogenic potential. The blood level reached 3840 mg/L [114]. This is about 100-fold higher than the blood alcohol level after excessive exposure to EBHR. In rabbits, no developmental toxicity could be detected up to the highest administered dose of 2370 mg/kg [115].

The risk of developmental effects from inhalation exposure during hand antisepsis is negligible, as indicated by experiments in rats [116]. No fertility or developmental effects were seen at inhalation exposures up to 16,000 ppm (30,400 mg/m^3^; [116–118]). The lowest reported No Observed Adverse Effect Level (NOAEL) for fertility by the oral route was 2000 mg/kg in rats, equivalent to a blood alcohol concentration of 1320 mg/L, although this was based on a significant increase in the number of small pups rather than a direct effect on fertility; such direct effects are not seen until much higher doses [119].

## Summary of risk assessment in the literature

Irvine [120] characterizes the situation as follows: “A specific NOAEL for human developmental toxicity of ethanol is unlikely to be determined from existing epidemiological studies because the results of all such studies are influenced to some extent by confounding nutritional and environmental factors and from the inherent imprecision in the assessment of ethanol exposure that is based on self-reporting. Developmental toxicity may result from drinking alcoholic beverages. This is not, however, considered relevant to the low blood alcohol concentrations resulting from any conceivable inhalation or dermal exposure in the workplace or through the directed use of any consumer product containing ethanol.”

The dermal adsorption of ethanol after hand rub use is below any toxic risk and is also lower than intake from non-alcoholic beverages. Furthermore, ethanol does not accumulate in the body. Concerning repeated dose effects, the lowest reported NOAEL is approximately 2400 mg/kg /day from a dietary study on rats [121]. Since 1959 [122], EBHR have been used in Europe for hand antisepsis and surgical hand preparation without any indication of toxic or reproductive toxicity side effects. It can be concluded from ethanol absorption data after exposure to EBHR that there is no evidence whatsoever that would justify classifying ethanol as a developmental reproductive toxicity category 2 under the EU CLP regulation, if used as intended for hand antisepsis.

## Occupational health assessment of ethanol

This may be divided into several categories as listed:

### Maximum workplace concentration (MAK value)

380 mg/m^3^ [123]. Pulmonary absorption of ethanol is most important in the workplace, while percutaneous absorption is of secondary importance [124].

### Acute toxicity

No local irritation occurs at ethanol concentrations up to 5000 ppm and no systemic effects occur up to 1000 ppm after many years of occupational experience [125]. In recent testing among volunteers, at 1000 ppm, no exposure-related changes in reaction time, choice response, or short-term memory were noted, nor was discomfort felt. First effects on the central nervous system (CNS) are expected only at much higher concentrations, producing blood alcohol levels in the range of 200 mg ethanol/L [125]. The blood alcohol levels achieved by exposures far above those related to hand antisepsis are on average more than 100-fold lower (Table 6).

When taken orally, CNS performance may already be impaired at blood levels of 200–300 mg ethanol/L; above 600–700 mg/L the CNS is significantly affected in the majority of people [125]. Thus, when EBHR is used for hand antisepsis, a resorption-related influence on the CNS can be absolutely excluded (Table 6).

### Chronic toxicity

No data are available on the effects of long-term inhalation exposure, although industrial workplaces where exposure opportunities exist are common.

Chronic consumption of large quantities of alcoholic beverages can lead to toxic effects on almost all organ systems. The liver is particularly affected, where damage initially manifests as fatty degeneration and can progress via necrotic and fibrotic stages to liver cirrhosis. The threshold value for triggering toxic liver damage is assumed to be 20–40 g/day for women and 60—80 g/day of ethanol for men with regular oral intake [125].

### Fetotoxic and teratogenic potential

The risk of fetal damage is unlikely provided the MAK value of 380 mg/m^3^ is not exceeded [126].

A teratogenic effect (alcohol embryopathy) has been demonstrated after oral intake of high doses. However, the ethanol concentrations in maternal blood at which these effects occur are of a magnitude not reached by inhalation exposure in the occupationally relevant concentration range. In animal studies, concentrations up to 20,000 ppm had no effect on the offspring, despite toxic effects on the mothers. A fertility-reducing effect and the influencing of sex hormone levels have likewise been demonstrated in humans and in animal experiments only after oral intake of high doses [126].

### Mutagenic potential

In animal studies, mutagenic effects were observed only at doses within the toxic range [126]. Since occupationally permissible ethanol concentrations do not significantly increase lifetime exposure, the mutagenic potential is considered negligible under these conditions.

### Carcinogenic potential

Long-term ingestion of large amounts of ethanol in the form of alcoholic beverages can cause tumors of the mouth, pharynx, larynx, oesophagus, liver, and possibly mammary glands and intestines. Since it has been shown that the lifetime internal exposure to ethanol from occupational exposure up to 500 ppm is still within the standard deviation of the endogenous exposure, it is estimated that exposure up to this limit does not contribute significantly to cancer risk [126].

The Poisindex® [127] provides the following classification for ethanol:

- TLV-TWA (Threshold Limit Value—Time-Weighted Average): Not listed.
- TLV-STEL (Threshold Limit Value–Short-Term Exposure Limit (TLV-STEL): 1000 ppm.
- TLV-C (Threshold Limit Value–Short-Term Exposure Limit (TLV-STEL): Not listed.
- Carcinogenicity Category: A3 (definition: confirmed animal carcinogen with unknown relevance to humans: the agent is carcinogenic in experimental animals at a relatively high dose, by route(s) of administration, at site(s), of histologic type(s), or by mechanism(s) that may not be relevant to worker exposure. Available epidemiologic studies do not confirm an increased risk of cancer in exposed humans. Available evidence does not suggest that the agent is likely to cause cancer in humans except under uncommon or unlikely routes or levels of exposure [128]).

An earlier published assessment with another type of classification came to an analogous conclusion and classified ethanol in category 5, defined “substances with carcinogenic and genotoxic effects, the potency of which is considered to be so low that, provided the MAK and BAT values are observed, no significant contribution to human cancer risk is to be expected. The classification is supported by information on the mode of action, dose dependence and toxicokinetic data pertinent to species comparison” [129].

It should be noted that these assessments were made for continuous occupational exposure during inhalation by handling of ethanol and are not comparable to the use of EBRH for hand antisepsis.

The values for acute and chronic toxicity or fetotoxic, teratogenic, mutagenic and carcinogenic effects refer exclusively to oral exposure. Initial effects are described at blood concentrations > 200 mg/L. Blood alcohol levels achieved with significantly higher-than-real-exposure by hand antisepsis, i.e., 10 applications of EBHR for 3 min each at 5-min intervals, were found to be considerably lower, with a maximum of 30.1 mg ethanol/L (Table 4). Thus, there is no indication of any acute or chronic hazard associated with the use of EBHR for hand antisepsis.

## Conclusion

Since 1977, the WHO has maintained a list of critical medications (WHO List of Essential Medicines), last updated in 2019. On that list, ethanol (70%, denatured) appears under antiseptics (15.1. Antiseptics) and under alcohol-based hand antisepsis agents (80% v/v; 15.2. Disinfectants) [130]. Ethanol is featured on the core list of basic-care active substances that should be available.

The doses of ethanol contained in EBHR and absorbed through the skin are in the range of those associated with the consumption of beverages with hidden ethanol content ≤ 0.5% v/v, such as apple juice and kefir, which are considered safe in accordance with EU Regulation No 1169/2011 (alcoholic content of beverages must be declared if it is higher than 1.2%). All studies support the conclusion that there is no significant risk of developmental or reproductive toxicity from repeated use of EBHR. Furthermore, the toxicological assessment concludes that dermal uptake of ethanol by health care workers or consumers would never result in exposure levels that are hazardous to health. Pires et al. [131] conclude “that the dermal and inhaled absorption of ethanol when using EBHR is minor and results in blood and urine concentrations comparable with other widely used alcohol-containing products of daily life.” The modeling approach of ethanol pharmacokinetics predicts that the consumption of one nonalcoholic beverage (estimated content of ethanol 0.5% vol/vol) would result in a peak blood concentration of 12 mg/L [107], an amount comparable with estimates resulting from intensive use of EBHRs. It should be remembered that EBHR can be produced locally from sugar cane, maize, manioc, rice, or several other natural by-products at a very low cost, using the procedure proposed by the World Health Organization, with extensive experience in many countries and health-care settings worldwide [5].

Hence, the WHO Task Force ABHR and KRINKO emphatically recommend maintaining ethanol as a biocidally active ingredient in hand rubs for use in healthcare settings. Ethanol used in EBHR is an effective and safe agent for the prevention of healthcare associated infections and spread of antimicrobial resistance. There are currently no alternatives to challenge the use of EBHR and we advocate continued use. If removed from our arsenal, we would lose a critical weapon against healthcare associated infections and risk a resulting increase in morbidity and mortality among patients worldwide.

## Data Availability

Not applicable.
